# Study of the internal mechanism of attention focus affecting countermovement jump performance based on muscle synergy theory

**DOI:** 10.1371/journal.pone.0306049

**Published:** 2024-07-25

**Authors:** Fan Peng, Dongxue Wang, Yonghao Zhang, Yongmin Xie

**Affiliations:** 1 Criminal Investigation Police University of China, Shenyang, China; 2 School of Strength and Conditioning Training, Beijing Sport University, Beijing, China; 3 Faculty of Health Sciences, Cancer Center, University of Macau, Macau SAR, China; 4 MOE Frontiers Science Center for Precision Oncology, University of Macau, Macau SAR, China; 5 Chinese People’s Liberation Army 91206 Force, Qingdao, China; University of Belgrade: Univerzitet u Beogradu, SERBIA

## Abstract

**Objective:**

The purpose of this study was to explain the internal mechanism of attention focus affecting performance of countermovement jump based on muscle synergy theory.

**Methods:**

Participants involved untrained group(N = 10) and high-level group(N = 11). Subjects performed countermovement jump with internal attention focus instruction (IF), external distal attention focus instruction (EDF), and external proximal attention focus instruction (EPF). The electromyography (EMG) signals of the dominant vastus lateralis muscle (VL), semitendinosus muscle (ST), tibial anterior muscle (TA), rectus femoris muscle (RF), and medial gastrocnemius (MG) were recorded. The non-negative matrix factorization was used to extract muscle synergy.

**Results:**

1) Attention focus did not affect countermovement jump performance and the number of muscle synergy in the high-level group (P>0.05). 2) Attention focus instructions affected the untrained group countermovement jump (P<0.05). and EDF and EPF reduced the number of muscle synergy. 3)The Cohen’s d of EDF (0.269) was less than EPF (0.377) in untrained group.

**Conclusion:**

For the untrained people, the improved motor performance caused by attention focus resembled the adaptive changes that occur with long-term training. The reason why an EDF is superior to EPF is that the former produces more thorough changes in muscle synergy.

## Introduction

Attention focus plays a pivotal role in optimizing motor performance, which is a concept extensively supported by empirical research [1–10]. It encapsulates the directed concentration of an individual’s attention towards specific aspects of movement execution. Attention focus was categorized into internal attention focus and external attention focus, the latter being further divided into external distal and external proximal focus. Internal attention focus pertains to concentrating on one’s own body movements, such as aiming to extend the knee as swiftly as possible. External proximal attention focus involves concentrating on a nearby point or object, such as attempting to leap beyond the start line as far as possible. In addition, external proximal attention focus refers to the practice of focusing attention on distant objects or markers, such as a cone placed far away when performing a jumping action. This phenomenon is often explained through the constrained-action hypothesis, which posits that external focus facilitates the natural self-organization of motor systems, thereby avoiding the restrictive effects of internal focus on automatic movement regulation [11,12].

Complex motor behavior, characterized by variations due to redundant degrees of freedom, is a manifestation of central nervous system processes that are modulated by factors such as joint and muscle dynamics as well as attentional focus during movement [13]. The muscle synergy theory facilitates the decoding of these intricate motor sequences, portraying them as amalgamations of modular motor elements. The central nervous system employs these elements in various manners to effectively regulate specific movements, thereby addressing the inherent complexity associated with coordinating multiple muscle groups [14,15]. The efficient motor execution emerges from the synergistic combination of these modules, which is a indispensable principle for mastering new skills and adapting movements. The synergistic combination of modules is evident in the of dynamic evolution of muscle synergies, the process of amidst skill progression and auxiliary interventions [16–20]. Though there are extensive researches on the relationship between attentional focus and muscle synergy, a comprehensive elucidation of how different types of external focus (proximal and distal) impact motor performance, particularly in terms of muscle synergy configuration, remains undecided.

This deficiency represented the catalytic premise of current investigation, which aimed to reveal the ways in which different attention focus can shape motor control tactics at the muscular synergy level. The study postulated that the modulation of attention focus will induce observable shifts in muscle synergies, thus reflecting the adaptive methods of central nervous system for optimizing movement. The purpose of research poised not only to enrich the existing constrained-action hypothesis but also to broaden the comprehension of neuromuscular adaptations that aid in the augmentation of motor performance. The study utilized non-negative matrix factorization algorithms to extract muscle synergy patterns from countermovement jump, which was a carefully chosen task due to its inherent safety and data collection practicability.

## Methods

### Participants

A total of 21 male participants were recruited for the study, including 10 individuals in the untrained group and 11 individuals in the high-level group. participants in the high-level group were required to reach the national level or above. The study considered the untrained group as Tier 0 and the high-level group as Tier 3 [21]. The specific information of participants was shown in Table 1. The training status were verified through the athlete technical information system of General Administration of Sport. In addition, participants were also required to be in good health, without any lower limb musculoskeletal diseases within the previous six months. Individuals in the untrained group were required to be in good health, without any lower limb musculoskeletal diseases within the previous six months, and without any prior systematic training or exercise. The study was followed the Declaration of Helsinki and was approved by the Ethics Review Committee of Beijing sport University (No. 2022181H). All participants had read the experimental instructions and informed consent was signed before the beginning of the experiment.

**Table 1:** Participant information.

	Year	Height (cm)	Weight (kg)	Training (year)
Untrained Group	20±1.3	177.4±6.5	65±4.3	0
High-level Group	20±1.4	177.1±7.4	73±7.5	7±1.3

### Procedures

Before the start of the experiment, the participant net weight was obtained by force plate (Kistler, model 9281CA). The dominant side of the participant was determined by the method of kickball [22]. Participants performed stationary kicks using the left and right sides. Participants should familiarize themselves with this movement before formal testing. For the formal test, each side performed 3 kicks and the results were recorded. The longest distance kicked by each side was selected for evaluation and the side with the longest distance kicked was determined to be the dominant side. Then the participants were required to perform preparatory activities, including warm-up on a power bicycle, static stretching, and dynamic stretching. During testing, the participants were guided by different attention focus instructions. The instruction including internal focus instruction (IF), external distal focus instruction (EPF), external proximal focus instruction (EDF), control group with no attention focus instruction (CF). The syntactic structure of the instruction language was similar, and the length of the instruction language is limited to 8–9 characters. Attention focus instructions were played to the participants through audio playback. The attention focus instructions is shown in Table 2.

**Table 2:** Attention focus instructions.

	Instruction
CF	When jumping, jump in your normal way.
EPF	When jumping, try to get as high as possible off the ground.
EDF	When jumping, try to get as close as possible to the ceiling.
IF	“When jumping, rapidly extend your hip and knee joints.

IF: Internal focus instruction. EDF: External distal focus instruction. EPF: External proximal focus instruction. CF: No instruction.

Prior to the countermovement jump test, participants were required to listen to the experimenter’s instructions. Each participant completed the no-instruction test first, and then a random number table was used to determine the test order of IF, EPF, and EDF. All participants completed three countermovement jump tests under each type of instruction, for a total of 12 countermovement jump tests. Participants performed countermovement jumps without arm swing (having their hands fixed on the hips) and squats down until the knees are bent at 90 degrees. The best of the three countermovement jumps took as the final result for each type of instruction. The participants rested 4 minutes between each type of instruction and 1 minute between each countermovement jump test. During the experiment, EMG signals of five dominant muscles, including the vastus lateralis muscle (VL), semitendinosus muscle (ST), tibialis anterior muscle (TA), rectus femoris muscle (RF), and medial gastrocnemius muscle (MG), were recorded at 2000 Hz using a Wireless EMG System (Trigno, Delsys, USA). The surface of the skin was shaved, prepared using an alcohol wipe (Boshile, CHN) with a mild abrasive. EMG electrodes affixed using adhesive tape (Delsys Trigno, USA), pressure sensitive adhesive tape (Hons, CHN) and cohesive bandages (Lefeke, CHN). The bipolar EMG sensors were placed center of each muscle belly under the guidance of therapists. Force–time data were collected with an in-ground two force plate (Kistler, model 9281CA) using a sampling rate of 1,000 Hz.

### Data and signal processing

**Fig 1:**
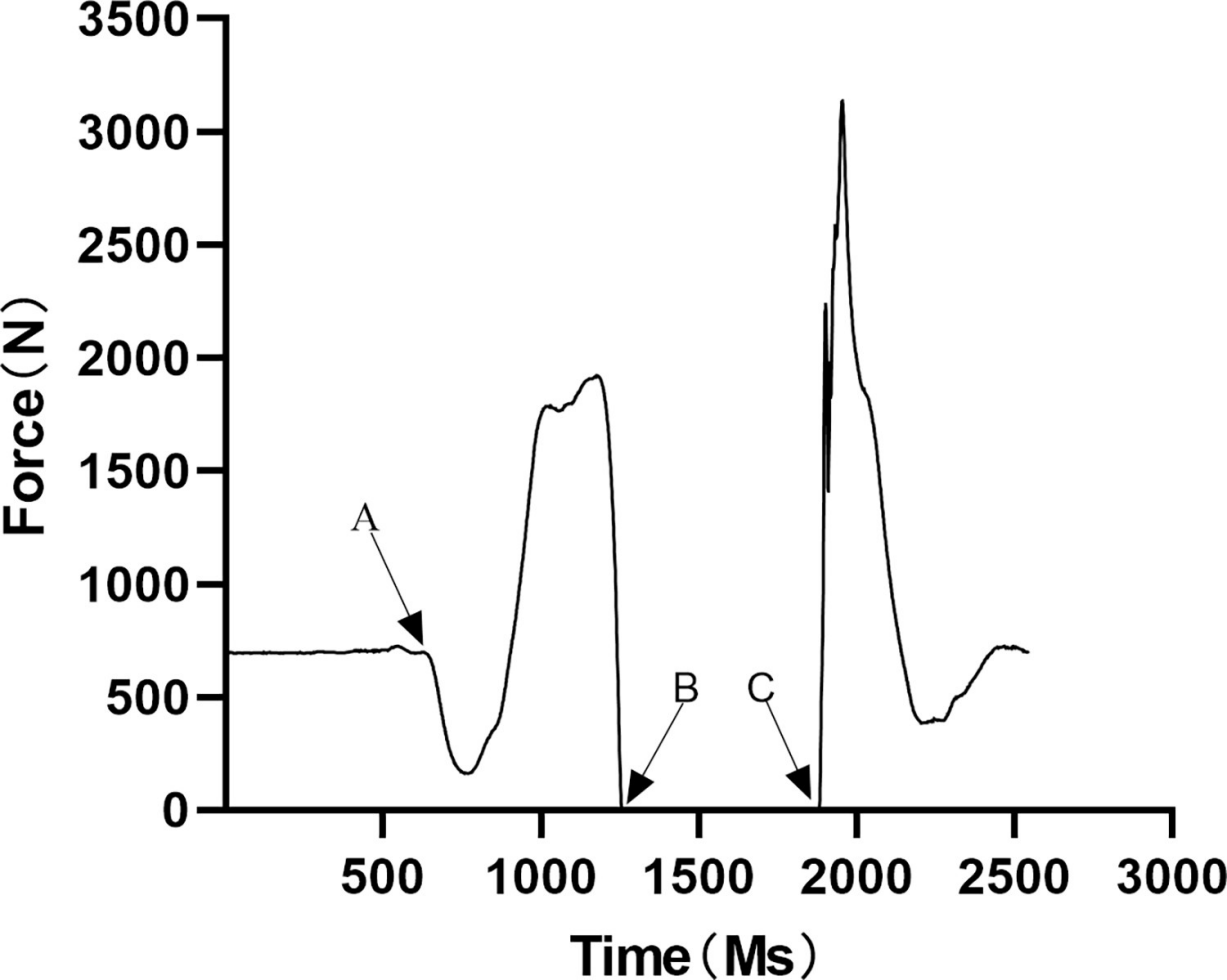
Force–time graph of a participant.

The data derived from force platform could be processed by Bioware software and exported to an Excel. Initiation of the jump movement was defined as the point where the force–time curve dropped below a threshold of 2.5% of body weight(A) [23], as show in Fig 1. The end of the jump and the start of the flight were calculated from when the force dropped to 10N(B), the end of the flight was calculated from when the force rise up to 10N(C) [24], as show in Fig 1. The countermovement jump performance could be calculated using the Impulse‑Momentum method [25], as shown in Eq (1). In this equation, J represented countermovement jump Impulse‑Momentum Calculus. *t*_*start*_ and *t*_*take−off*_ were the time at instant of the propulsion phase and take-off (A-B), respectively. The *F*_*vGRF*_ and *F*_*g*_ were the vGRF and the body mass of the participant, respectively. g represented universal gravitational constant.


$$J=\int_{t_{start}}^{t_{take-off}}\left(F_{vGRF}-F_{g}\right)dt$$ (1)

The EMG data was exported to the excel by EMG works Analysis and pre-processed by MATLAB R2021a. The range of EMG data could be extracted from the start of countermovement jump to the take-off (A-B, as show in Fig 1). After extracting the EMG signals, a notch filter (50 Hz) can be used to remove power-line interference, followed by a 4th order band-pass filter (20–500 Hz) which could filter out unwanted frequencies, the rectified signals can be passed through another 4th order low-pass filter (20 Hz) for further noise reduction [26–28]. Using the RMS method, envelope lines could be extracted from the filtered signal. After filtering, the EMG amplitude was normalized.

### Muscle synergy extraction

MATLAB R2021a and non-negative matrix factorizationwere used to extract muscle synergy from the EMG signals, as shown in Eq (2). Muscle synergy model reference Synchronous muscle synergy.


$$V_{M\times N}=[EMG_{1}\;EMG_{2}\;EMG_{3}\ldots EMG1_{M}{]}^{T}\approx {\left(WH\right)}_{M\times N}=\sum_{i=1}^{P}\;W_{M\times P}\;H_{P\times W}+e=V_{M\times N}^{\prime }$$ (2)

In this equation, *V* represented the raw EMG matrix, where *M* denoted the number of surface EMG channels and *N* refered to the number of samples. *P* was defined as the number of muscle synergy obtained through non-negative matrix factorization, with *M≥P≥1*.The *W*_*M*×*P*_ was used to represent the muscle-synergy vector, while the *H*_*P*×*W*_ represented time-varying activation coefficients. The reconstructed matrix, $V_{M\times N}^{\prime }$, was obtained by multiplying the *W* and *H* matrices, along with a residual error term, *e*. The reconstruction quality was determined by the variance accounted for VAF, as shown in Eq (3).


$$VAF=1-\frac{{\sum_{i=1}^{T}\left(V-V\prime \right)}^{2}}{{\sum_{i=1}^{T}\left(V-mV\right)}^{2}}$$ (3)

The original EMG matrix was denoted as *V*, with *mV* representing the mean of the envelope of the *i-the* muscle in the *V* matrix. The reconstructed matrix was represented by V. The *V*′ matrix was iteratively reconstructed using a matrix factorization approach, and the reconstruction accuracy was evaluated using the VAF value. Iterations were stopped when the VAF value changes by less than 0.01% for 20 consecutive iterations, indicating the convergence of the algorithm [29]. Since the *V*′ matrix started from a random initialization at each iteration, the minimum number of muscle-synergy vector required to achieve a VAF value of at least 0.9 for ten consecutive trials was used as the threshold number of muscle-synergy vector for both the high-level and normal-level groups [30].

### Muscle synergy similarity and merging

The merger of muscle-synergy vector required matching and calculating the similarity between different muscle-synergy vectors [16,30].

Each participant’s muscle-synergy vectors in CF was matched with the muscle-synergy vectors in IF, EDF and EPF. Muscle-synergy vector matching was performed using scalar product. The matching process was shown in Eqs (4)–(6).


$$X_{v}=\frac{V_{i}\cdot V_{j}}{\left|V_{i}|\cdot |V_{j}\right|}\;i,j=(1,2,3,4\ldots \;\ldots K)$$ (4)

$$N(i)=max[X_{v}(1,i),\;X_{v}(2,i),\;X_{v}(3,i)\ldots \;\ldots \;..X_{v}(K,i)]\;i=(1,2,3,4\ldots \;\ldots K)$$ (5)

$$C_{v}\;(i)=X_{v}\;\{N(i),\;i\}\;i=(1,2,3,4\ldots \;\ldots K)$$ (6)

In Eq (4), *V*_*i*_ represented muscle-synergy vector in CF, *V*_*j*_ represented the matching muscle-synergy vector, |*V*_*i*_| and |*V*_*j*_| represented the magnitudes of the *V*_*i*_ *and V*_*j*_ vector. Eq (4) calculated the similarity between *V*_*i*_ and *V*_*j*_, with a similarity range of 0.000–1.000. 0.000 being the minimum similarity and 1.000 being the maximum similarity. *X*_*v*_ is a *K*×*K* proximity matrix. Eqs (5) and (6) described the matching process. The max function assigned the highest similarity value in each row of the *K*×*K* matrix to the corresponding row of the *N(i)* matrix. *N(i)* was the row number K, and *C*_*v*_(*i*) assigned the i-th muscle-synergy vector |*V*_*j*_| of the K rows to match the reference matrix |*V*_*i*_|.

**Fig 2:**
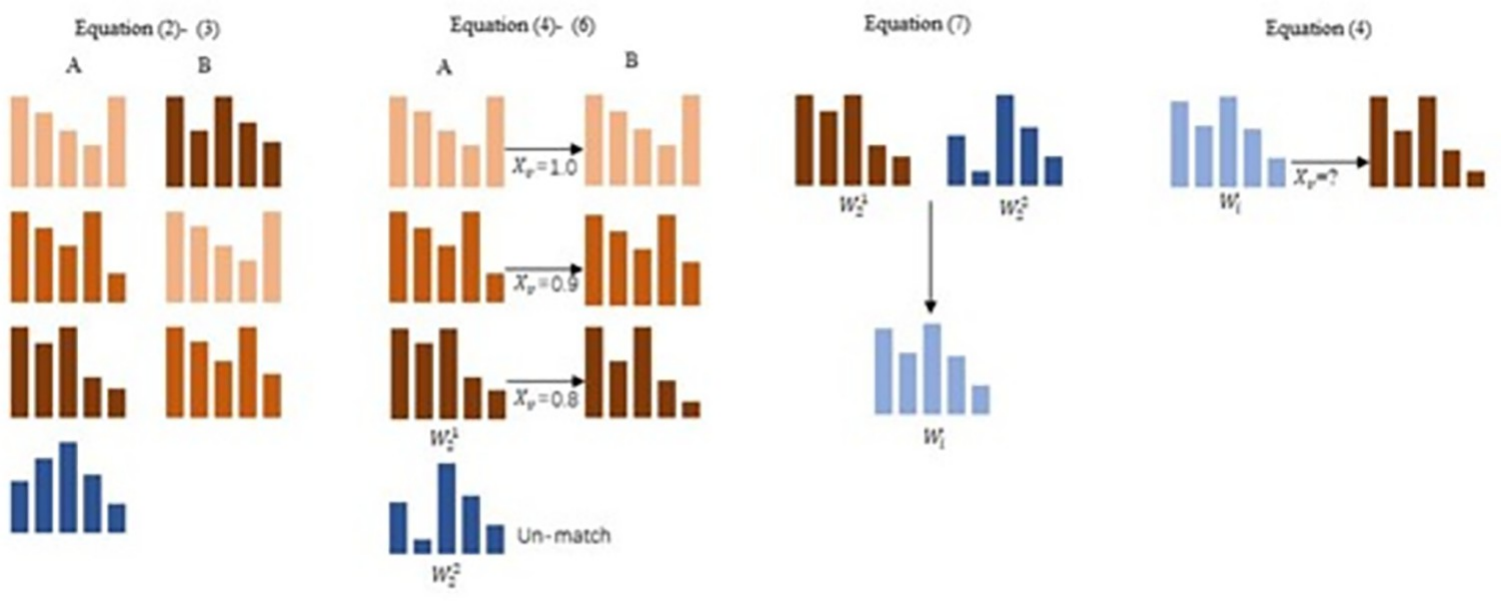
The procession of muscle synergy similarity and merging.

The muscle synergy of merging was shown in Eq (7). In Eq (7), *W*_*i*_ represented the decomposition muscle-synergy vector of *V*_*i*_, and $W_{K}^{B}$ represented the merged muscle-synergy vector. *B* represented the number of merged matrices, and $m_{k}^{i}$ represented the contribution value of $W_{K}^{B}$ to *W*_*i*_. When $m_{k}^{i}$ ≥ 0.02, it was considered as the minimum contribution value threshold. The $m_{k}^{i}$ was calculated using non-negative least squares method, which was implemented using the function lsqnonneg in MATLAB R2021a. The muscle-synergy vector of the lowest similarity in CF was considered as $W_{K}^{B}$. The merged muscle-synergy vector, defined as *W*_*i*_, was calculated for its scalar product with the muscle-synergy vector that was the lowest similarity in EDF or EPF. The procession as show in Fig 2.


$$W_{i}\approx \sum_{K}^{B}m_{k}^{i}W_{K}^{B}\;m_{k}^{i}\geq 0,\;K=1\ldots \;\ldots B$$ (7)

### Statistical analysis

The acquired kinematic and muscle synergy data were processed using SPSS22 statistical software, presented as mean ± standard deviation. Two-factor repeated measures ANOVA and Bonferroni correction analysis were used to compare the differences in countermovement jump Impulse‑Momentum between the high-level group and untrained group under different attention focus instructions. The Friedman test was used to analyze the differences in the number of muscle-synergy vectors between the high-level group and untrained group under different attention focus instructions and conduct a post-mortem examination. The effect size Cohen’s d was calculated for the similarity before and after mergence. A “*p* < 0.05” was considered statistically significant.

## Result

### The Impulse‑Momentum of countermovement jump

The sphericity test showed *P* = 0.001, and the within-participants effects test (Greenhouse-Geisser) indicated that there were significant differences in the effects between the instructions (*P* = 0.002, F = 6.077, η^2^ = 0.242), and the interaction effect between instruction and group was significant (*P* = 0.062, F = 2.863, η^2^ = 0.127).

Analysis showed no significant differences between attention focus instructions in high-level group (*P* = 0.720, F = 0.452, η^2^ = 0.074). Bonferroni correction analysis showed that there were no significant differences between CF and IF (*P* = 0.166, SE = 3732.010), CF and EPF(*P* = 0.166, SE = 3258.684), or CF and EDF (*P* = 0.166, SE = 3267.195). There were also no significant differences between IF and EPF (*P* = 1.666, SE =, 3892.203) and between IF and EDF (*P* = 0.166, SE = 3970.013). Additionally, EPF and EDF had no significant differences (*P* = 0.166, SE = 2313.649).

There were significant differences between attention focus instructions in untrained group (*P* = 0.004, F = 6.504, η^2^ = 0.534). Bonferroni correction analysis showed that there were no significant differences between CF and IF (*P* = 0.166, SE = 3944.719), but there were significant differences between CF and EPF (*P* = 0.019, SE = 3534.061) and between CF and EDF(*P* = 0.000, SE = 3557.969). There were also significant differences between IF and EPF (*P* = 0.033, SE = 4056.550) and between IF and EDF (*P* = 0.001 SE = 3933.663). Additionally, EPF and EDF had significant differences (*P* = 0.029, SE = 2302.601). The countermovement jump Impulse Momentum Calculus and variable under each condition is shown in Tables 3 and 4.

**Table 3:** The countermovement jump Impulse Momentum Calculus.

attention focus	High-level Group	Untrained Group
CF	218396±22861	170785±33274
IF	216094±24045	170531±34636
EPF	218591±24256	179807±31406*
EDF	220274±28558	185239±31350*

*: *P*<0.05 when compared with the CF group.

**Table 4:** Mean±SD scores for each countermovement jump variable under each condition.

Group	Attention focus	Peak GRF(N)	Relative peak GRF(N·kg-1)	RSI
Untrained Group	CF	1608±333	24.843±2.566	0.706±0.103
	IF	1607±334	24.848±2.465	0.717±0.127
	EPF	1611±311	24.958±2.284	0.756±0.157
	EDF	1623±312	25.191±2.682	0.764±0.190
High-level Group	CF	1854±222	26.186±1.544	0.775±0.128
	IF	1903±175	26.944±1.300	0.806±0.087
	EPF	1882±253	26.539±1.483	0.801±0.096
	EDF	1853±200	26.172±0.773	0.798±0.135

### The number of muscle synergy

**Fig 3:**
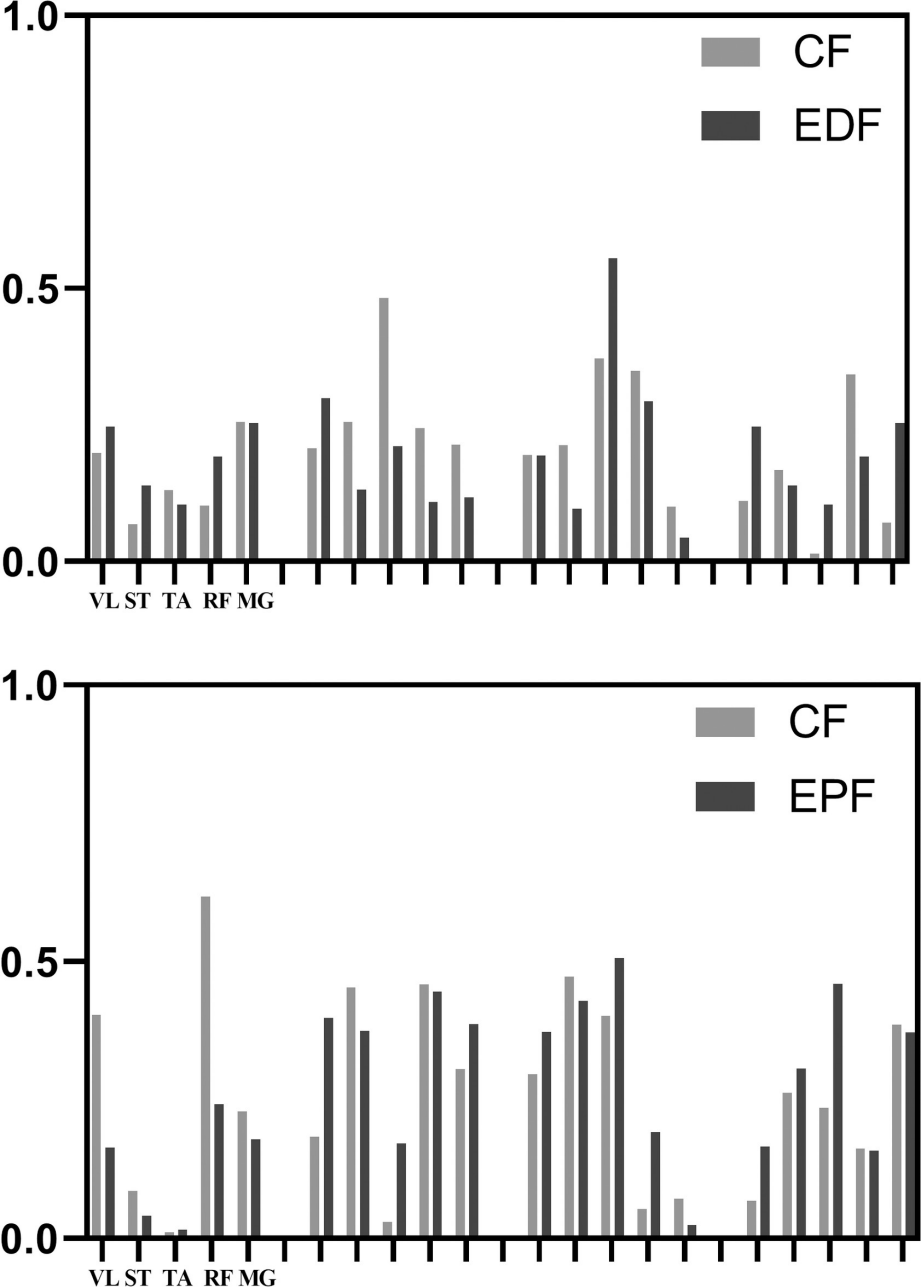
The same number of muscle synergy.

**Fig 4:**
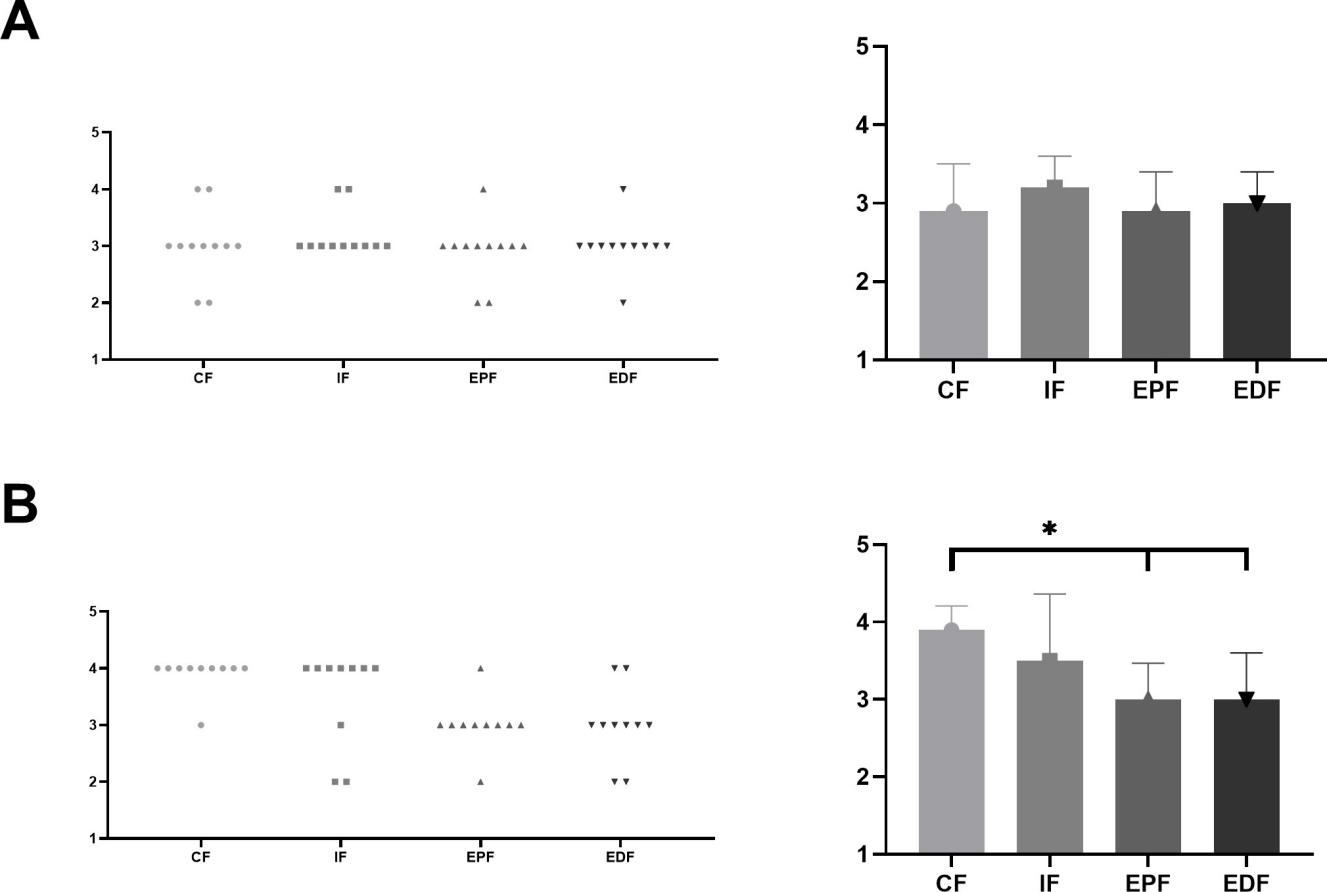
Attention focus effect of on the number of muscle synergy.

In the high-level group, attention focus did not affect the number of muscle synergy (*P* = 0.553). In the untrained group, attention focus had a significant effect on the number of muscle synergy (*P* = 0.000). Bonferroni correction showed that there were no significant differences between CF and IF groups (*P* = 0.823), while there were significant differences between CF and EPF groups (*P* = 0.008) and CF and EDF groups (*P* = 0.023). There were no significant differences between IF and EPF (P = 0.497) and IF and EDF (*P* = 0.962), and no significant differences between EPF and EDF (*P* = 1.000). An example of the constant number of muscle synergy under CF-EPF and CF-EDF in the untrained group was shown in Fig 3. The changes in the number of muscle synergy under different attention focus instructions were presented in Fig 4.

**Fig 5:**
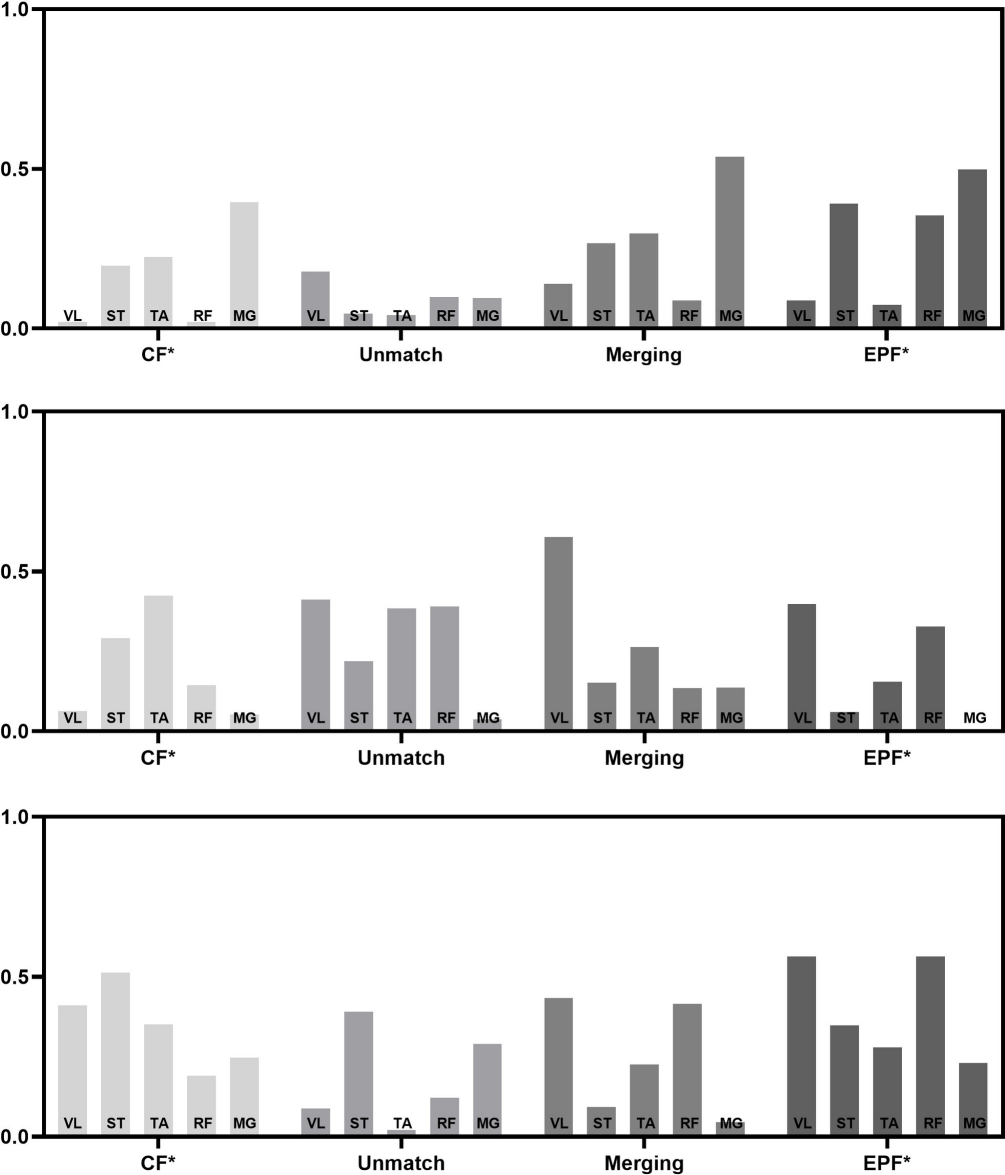
The merger of muscle synergy in EPF.

**Fig 6:**
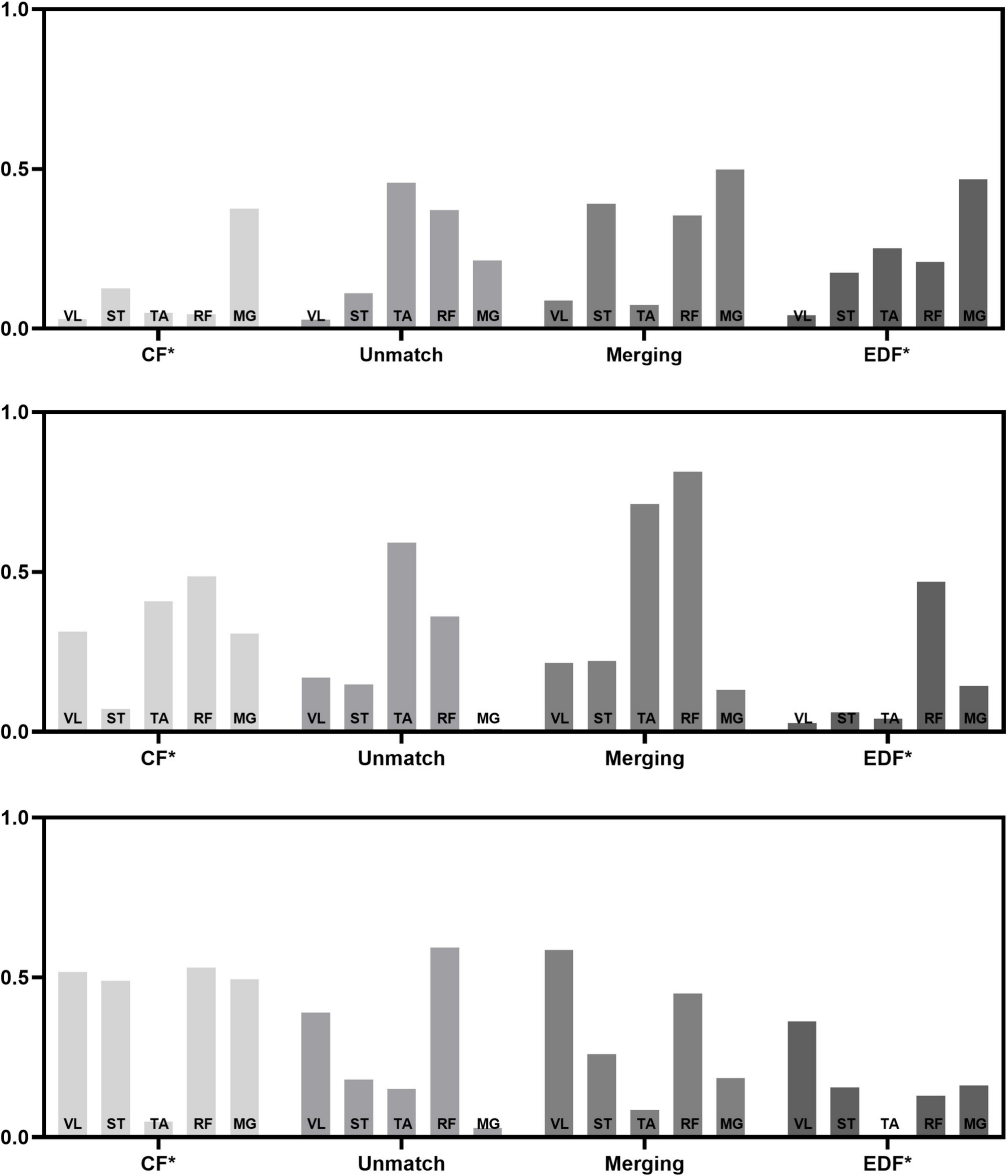
The merger of muscle synergy in EDF.

In the untrained group, we noted a decrease in the number of muscle synergy (from CF to IF; EPF and EDF). The merger of muscle-synergy vector mainly involved with the combination of two muscle-synergy vectors (CF) to form a new one. There were 16 merging muscle-synergy vectors, with 8 in the EPF group and 8 in the EDF group, as shown in Figs 5 and 6. The average similarity of the muscle-synergy vectors before merging in the EPF group was 0.774±0.148. After merging, it was 0.828±0.139. The average similarity of the muscle-synergy vectors before merging in the EDF group was 0.771±0.09. After merging, it was 0.797±0.102. The mergence effect in the EPF group (0.377) was higher than that in the EDF group (0.269). The similarity before and after muscle synergy mergence is shown in Table 5.

**Table 5:** Muscle synergy similarity.

	Before merge	After merge	Cohen’s d
EPF	0.774±0.148	0.828±0.139	0.377
EDF	0.771±0.091	0.797±0.102	0.269

## Discussion

In high-level group, attention focus had no effect on the number of muscle synergy and the performance of countermovement jump. In untrained group, EDF and EPF could significantly increase the performance of countermovement jump and decrease the number of muscle synergy. The EDF decomposed muscle-synergy vector more thoroughly than the EPF. This implies that change in muscle synergy and countermovement jump performance were associated with attention focus in the untrained group.

Research found that external attention focus instructions (EF) were superior to IF in the athletes or Tier 2 [2,31]. However, these studies have poor similarity in the syntax and structure of instructions. For example, the IF included three cues: push legs, swinging arms, and raising knees, but the EF included two cues: get off the starting blocks and cross finish line [31]. Due to the dissimilarity in syntax and cue, there were some conflicting findings. For example, the IF was that focus on driving your legs back explosive and EF was focus on driving the ground back as explosively as possible [32]. There were research findings which were relatively consistent in untrained people, indicating that EF was superior to IF [10]. On the external distal attention focus and external proximal attention focus, most of the studies had focused on athletes. Research suggested that EDF was superior to EPF [33,34]. However, these instructions varied greatly in the allocation of attention resources. Although the instructions of one study were reasonably designed, the training status of the participants could not be considered athletes [35]. Therefore, the effectiveness of attention focus was influenced by syntax and the cues of instructions [36,37].

The constrained-action hypothesis suggested that attention focus may constrain automation. In subsequent studies, the hypothesis was tested based on the movement performance and the fluency of movements [38–40]. These studies only validated the hypothesis, but the hypothesis still could not explain the differences between external distal attention focus and external proximal attention focus. For the high-level group, the number of muscle-synergy vectors remained stable under the CF, IF, EPF, and EDF. This suggested that attention focus does not affect the automation process of the high-level group participants. Automation could be understood as the fixed sequence of actions, the unconscious completion of the actions, and the consistency of the actions [41]. The automation of movements was associated with the stability of the muscle synergy. For example, the muscle-synergy of athletes remain basically the same when athlete completed a special movement [42]. Similar muscle synergy was also observed when exercising on a treadmill at different speeds [43]. countermovement jump were highly automated for high-level group participants. Automation means that individuals will continue to follow stable movement patterns if no novel stimuli are emerged. In long-term training, athletes have received many instructions similar to IF, EDF, EPF, so when athletes receive IF, EDF, EPF, there was no movement variation and no need to invest additional attention resources [44]. Conversely, when the automatic movements encounter novel stimuli, muscle synergy and movement performance will change. For example, when a dancer walks on a balance beam, the number and structure of the dancer’s muscle synergies change, requiring more time than normal walking [17].

For the untrained group, there were more muscle synergies under the CF and IF than the EPF and EDF. EDF decomposed muscle-synergy vector more thoroughly than the EPF (0.269 < 0.377). Researchers found that untrained individuals have more muscle synergies than athletes [16]. Muscle synergistic vetor merging is often seen in stroke patients [45,46]. Although the participants and interventions of these studies were different, the participants’ exercise capacity was accompanied by the changes in the muscle synergy. This was similar to this study, in which participants receiving EDF and EPF improved their countermovement jump performance and changed their muscle synergy vectors. Changes in muscle synergy or motor variability during growth or initial motor skill learning allows individuals more space for exploration [47]. Attention focus instructions were often associated with correct technical movements. It was difficult for untrained people to understand IF. Since the untrained people may not know the specific way of human joint movement or understand the proper noun. This made individuals increase the investment of attention resources thus limiting the automation process. EDF and EPF involved only approaching or moving away from a goal, which is easier for untrained groups to understand. As a result, the participant’s mode of action guides the development in the right direction. In order to accomplish more challenging tasks, individuals adapted existing muscle synergy to meet the needs of new tasks [48]. This explained the degree of similarity in muscle synergy before and after the merger. The combined similarity of EPF was lower than that of EDF, indicating greater motor variation from EPF and EDF. Correct motor variation associated with improved athletic performance, which may be why EDF is superior to EPF. EDF allows individuals to walk farther on the right path. If the coach gives proper verbal feedback, an athlete’s incorrect movement could be corrected quickly. The given verbal feedback could be viewed as attention focus [10]. It was due to the fact that feedback was directional, such as tarting with an anterior body lean. The differences between high-level group and untrained group might be due to long-term training, where athletes used the same muscle synergy to adapt to different situations [49]. This movement strategy allowed the central nervous system to use fewer motor modules to handle a variety of sport tasks [50]. Participants in the untrained group had fewer sports modules, were incompatible or maladaptive to the new task, and needed to adjust their muscle coordination to accomplish the task [51]. Whether it was humans or mammals, generating new muscle synergies or decomposing and merging existing muscle synergies is a basic strategy to improve physical ability [52]. This was similar to how certain muscle-synergy vector are merged under the CF to enhance the performance of countermovement jump in this study.

## Conclusion

For the untrained group, the improved motor performance caused by attention focus resembled the adaptive changes that occur with long-term training. The reason why an external distal attention focus was superior to an external proximal attention focus is that the former produces more thorough changes in muscle synergy.

## Limitation

There were also some shortcomings in this study. Muscle synergy consists of time-varying activation coefficients and muscle-synergy vector. The optimal approach for this study would be to simultaneously analyze the changes in time-varying activation coefficients and muscle-synergy vector before and after applying attention focus. In addition, it was important to analyze fused temporal patterns and coordination units simultaneously. Unfortunately, there is no prove method available for fusing temporal patterns. Therefore, this was unable to determine which coordination unit has the greatest effect on countermovement jump, as well as the different roles of coordination units at different time points. It was hoped that more mature algorithms could be developed to address this issue in the future.

## Supporting information

S1 File (DOCX)

S2 File (PDF)
