# Supplementary material for: Study of the internal mechanism of attention focus affecting countermovement jump performance based on muscle synergy theory
Source: PLoS One. 2024 Jul 25;19(7):e0306049. doi: 10.1371/journal.pone.0306049 (PMC11271881; doi:10.1371/journal.pone.0306049)

**Authors Change Statement**

Title: Study of the internal mechanism of attention focus affecting countermovement jump performance based on muscle synergy theory.

Manuscript number：PONE-D-23-41204R1

Authorship list:

Fan Peng^1^ Dongxue Wang^2^ Yongmin Xie^3^*

*1* *Criminal Investigation Police University of China, Shenyang, China.*

*2 School of sports medicine and rehabilitation, Beijing Sport University, Haidian, China.*

*3 School of Strength and Conditioning Training, Beijing Sport University, Haidian, China.*

**Corresponding author:*

*Yongmin Xie*

*xieyongminbsu@163.com*

Final author list:

Fan Peng^1,4^ Dongxue Wang^2a,3^  Yonghao Zhang^2b^ Yongmin Xie4^*^

*1* *Criminal Investigation Police University of China, Shenyang, China.*

*2a Cancer Center, Faculty of Health Sciences, University of Macau, Macau SAR, China.*

*2b Chinese People's Liberation Army 91206 Force, Qingdao, China*

*3 MOE Frontiers Science Center for Precision Oncology, University of Macau, Macau SAR, China*

*4* *School of Strength and Conditioning Training,* *Beijing Sport University, Beijing, China.*

****Corresponding author:*

*Yongmin Xie*

*xieyongminbsu@163.com*

We did not add Yonghao Zhang as an author at the time of submission because of the discipline of the Chinese People's Liberation Army (PLA) and the lack of supervision on Fan Peng and me, as explained in the Request for Authorship Change(s). The Author’s details with the following:

Given/First Name:*Yonghao

Middle Name:

Family/Last Name:*Zhang

E-mail Address:*1650815249@qq.com

Institution:*Chinese People's Liberation Army

Department:*91206 Force

Zip or Postal Code:*266000

Country or Region:*China

Contributor Roles:*Software

Dongxue Wang graduated from Beijing Sport University on June 12, 2024, and went to the University of Macau to pursue his PhD. We have therefore changed Dongxue Wang's affiliation.

Fan Peng graduated from Beijing Sport University in June 2023 and went to work for Criminal Investigation Police University of China. Funding support for this paper was jointly applied by Fan Peng and me at Beijing Sport University, so Fan Peng needs to add affiliations.

Finnally, I appreciate you sticking with me.

Signed:


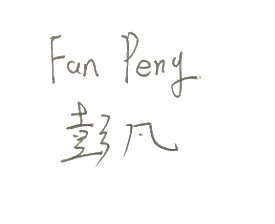

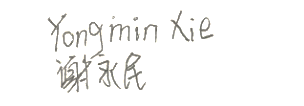

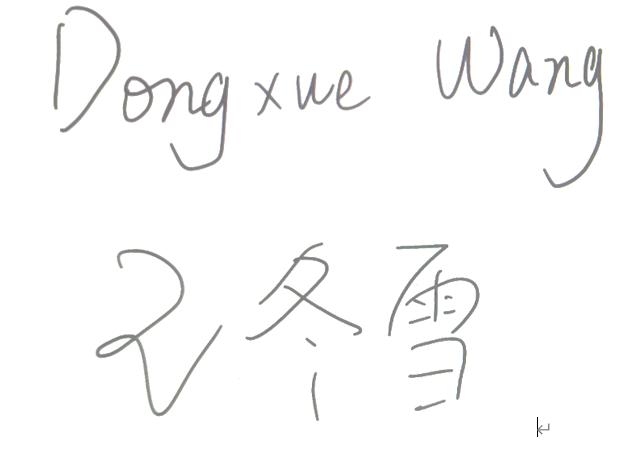

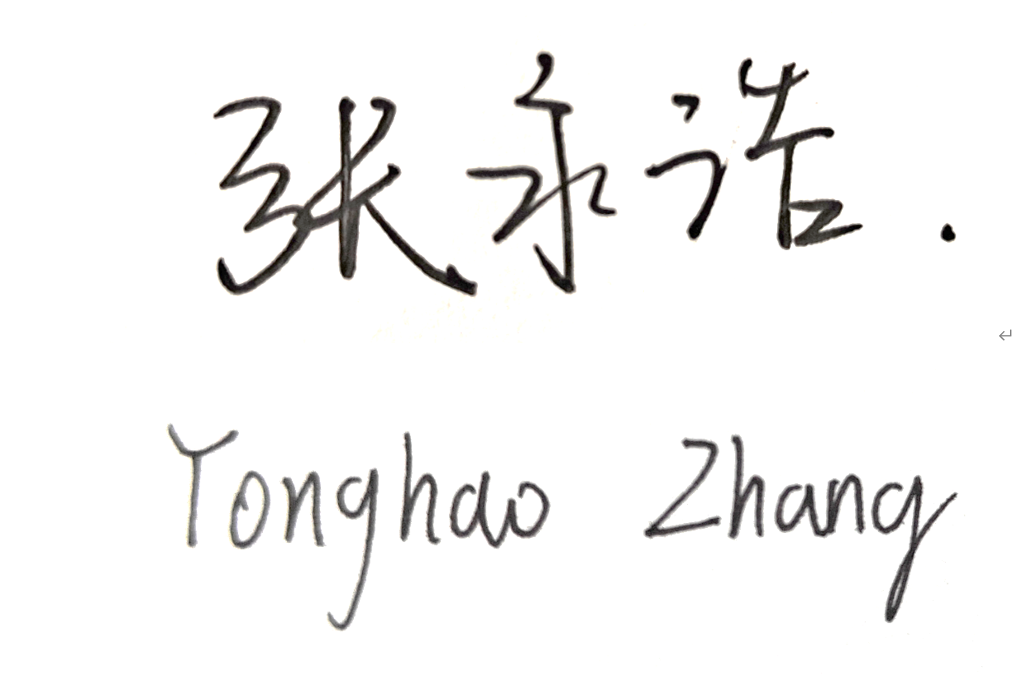

Supplement: S1 File — (DOCX) [file pone.0306049.s001.docx]
